# Assessment of Ecological Risk of Heavy Metal Contamination in Coastal Municipalities of Montenegro

**DOI:** 10.3390/ijerph13040393

**Published:** 2016-03-31

**Authors:** Boban Mugoša, Dijana Đurović, Mirjana Nedović-Vuković, Snežana Barjaktarović-Labović, Miroslav Vrvić

**Affiliations:** 1 Institute of Public Health of Montenegro DžonaDžeksona bb, 81000 Podgorica, Montenegro, boban.mugosa@ijzcg.me (B.M.); mirjana.nedovic_vukovic@ijzcg.me (M.N.-V.); 2 Health Centre of Bar, JovanaTomaševića 42, 85000 Bar, Montenegro; montelabovic@t-com.me; 3 Faculty of Chemistry, University of Belgrade, Studentski trg 12-16, 11000 Belgrade, Serbia; mmvchem@sezampro.rs

**Keywords:** pollution, soil, urban parks, ecological risk index

## Abstract

Assessment of heavy metal concentrations in the soil samples of urban parks and playgrounds is very important for the evaluation of potential risks for residents, especially children. Until recently, there has been very little data about urban parks pollution in Montenegro. To evaluate the sources of potential contamination and concentration of heavy metals, soil samples from coastal urban parks and kindergartens of Montenegro were collected. Based on the heavy metal concentrations, multivariate analysis combined with geochemical approaches showed that soil samples in coastal areas of Montenegro had mean Pb and Cd concentrations that were over two times higher than the background values, respectively. Based on principal component analysis (PCA), soil pollution with Pb, Cd, Cu, and Zn is contributed by anthropogenic sources. Results for Cr in the surface soils were primarily derived from natural sources. Calculation of different ecological contamination factors showed that Cd is the primary contribution to ecological risk index (RI) origins from anthropogenic, industry, and urbanization sources. This data provides evidence about soil pollution in coastal municipalities of Montenegro. Special attention should be paid to this problem in order to continue further research and to consider possible ways of remediation of the sites where contamination has been observed.

## 1. Introduction

Recently, urban soils have become highly influenced by anthropogenic activity [1] due to rapid urbanization and industrialization. Urban soils represent a “sink” of heavy metals from different sources of pollution such as: vehicle emissions, industrial wastes, dust sedimentation, coal combustion, precipitation, and other activities [2]. In the past few decades there have been several studies about heavy metal content in urban soils in many cities such as Palermo, Madrid, Hong Kong, Naples, Sevilla, Belgrade, Kavala, *etc.*—the first studies even started between 1960–1970 [3,4,5,6,7,8,9]. The biggest problem with heavy metals is the fact that they are persistent, and it is very difficult to eliminate them from the environment [10]. Heavy metals can exert their toxicity via dermal, inhalation, and ingestion pathways, from urban soils, and influence human health with severe consequences [11].

Humans are exposed to soil contamination through three different pathways; inhalation, ingestion, and dermal contact (skin exposure). Urban children spend most of their free time in parks and playgrounds, and they are frequently exposed to soil. They can ingest a significant amount of soil, between 39 and 270 mg⋅kg^−1^, due to their typical hand-to-mouth behavior, especially up to the age of six [12,13]. In parks, playgrounds, and residential areas, urban soils (*i.e.*, soils which are not used for agriculture purposes) can influence children’s health—due to their higher sensitivity, children are at a higher risk of exposure to the toxic metals than adults. Many geochemical approaches, such contamination factor (CF), ecological risk factor (Er), ecological risk index (RI), and geochemical index methods, are used for evaluation of anthropogenic influence on urban soil, and many studies were performed using these approaches. Ecological geochemistry assessment is very simplified using this calculation of pollution indices. These indexes evaluate the degree of pollution in soils, and help in the interpretation of soil quality [14,15,16,17,18,19].

Principal component analysis (PCA) as a multivariate chemometric technique is usually used as an additional method of heavy metal monitoring [20,21,22]. In this study, the results of Pb, Cd, Cu, Zn, and Cr concentrations in the soil samples from parks and playgrounds of coastal municipalities of Montenegro, as well as the calculation of contamination factors, ecological risk factors, the potential ecological risk index, and the index of geo-accumulation were performed. There is almost no data of urban soil quality in Montenegro. The authors would like to emphasize the fact that this kind of coastal soil quality research was performed for the first time in Montenegro. Using multivariate statistical methods in combination with wide a range of indices represents a novel approach for assessing the distribution of metals in urban soils which can be applied to other similar contaminated soil systems. 

## 2. Materials and Methods

### 2.1. Reagents and Standards

Analytical grade chemicals were used throughout the study. There was no further purification for the preparation of all reagents and calibration standards. Deionized ultra pure water was used with conductivity <1 µS⋅cm^−1^. A certified metal stock solution of 1000 mg⋅L^−1^ (J.T. Baker) by successive dilution with deionized water was used for preparing standards for calibration.

### 2.2. Sampling and Metal Analysis

**Figure 1:**
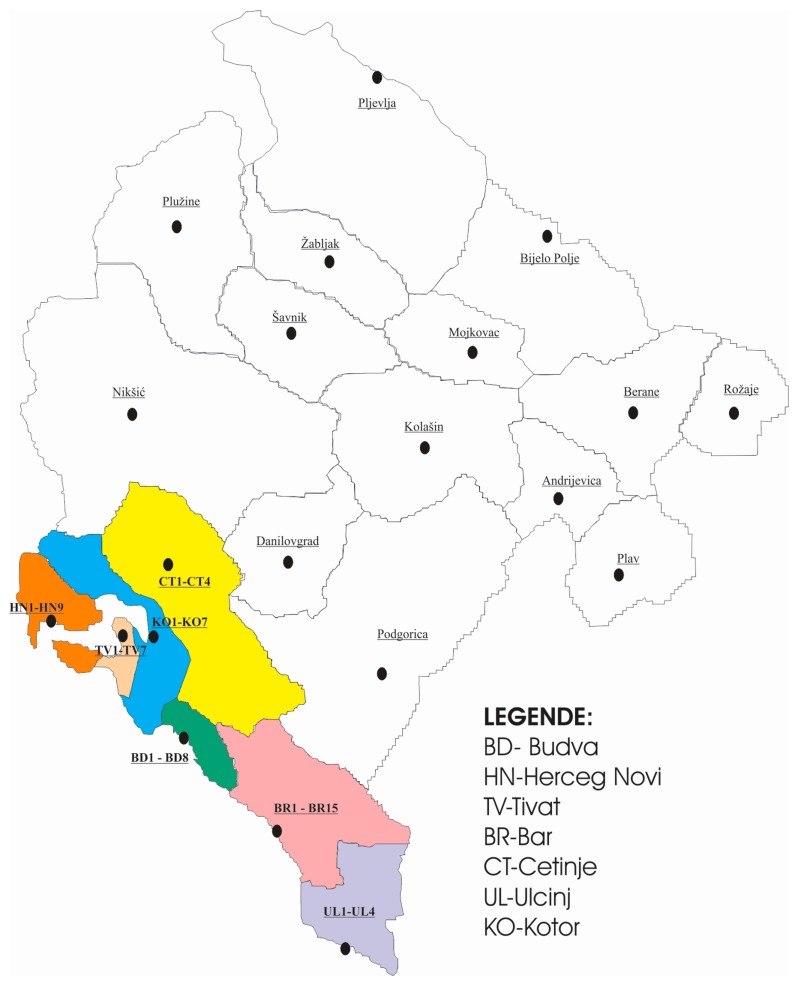
The map of a study area with sampling locations.

Montenegrin Coast covers the narrow coastal strip of the Oštro peninsula (Croatia) to the mouth of the Bojana River (on the border with Albania) and the Kotor Bay. The coastline is 293.5 km long. This study evaluated the concentration levels of five toxic metals—Pb, Cd, Zn, Cu, and Cr—from surface soil samples taken from the playgrounds in public parks and kindergartens in coastal municipalities of Montenegro (Ulcinj, Bar, Budva, Kotor, Tivat, Herceg Novi and Cetinje). Sampling sites are shown in Figure 1 and cover 2501 km^2^ of total Montenegrin area. Fifty-four soil samples, that represent the total number of city parks and kindergartens in this area, were studied. Coordinates and areas of sampling sites, as well as soil classification in accordance with national and WRB (World Reference Base), are given in Table 1 [23,24].

Sampling was conducted during October and November, 2014. Approximately 500 g of soil samples from the top 10 cm layer, within a 20 cm × 20 cm of surface soil, consisting of three sub-samples, were taken and mixed to obtain a bulk composite sample at each playground. Sampling was conducted on nearby playground equipment, such as swings, slides, *etc.* After sampling with a stainless trowel, samples were transferred to the laboratory in plastic bags. Foreign objects and stones were hand-removed, and the samples were air-dried for several days. Samples were gently crushed and sieved to 2 mm, and 1.0 ± 0.01 g was weighed for analysis after drying at room temperature. U.S. EPA 3052 method for microwave acid digestion was used for the sample preparation [25]. The concentrations of Pb and Cd were determined by graphite furnace atomic absorption spectrometry (GFAAS) (240Z AA Agilent Technologies-Netherlands, Santa Clara, CA, USA) because this method allowed more workable values for Pb and Cd due to a lower detection limit compared to ICP-OES. Zn, Cu, and Cr were determined by inductively coupled plasma-optical emission spectrometry (ICP-OES) (AMETEC-Spectro Arcos, Germany). Each sample was carried out in triplicate.

### 2.3. Assessment of Soil Contamination

The assessment of soil contamination was based on the calculation of the following factors: contamination factor (CF), ecological risk factor (Er), potential ecological risk index (RI), and index of geo-accumulation (Igeo). The ratio between the total metal content in soil (Cs) and the normal concentration levels (background concentration Cb) [26] was used for the CF calculation, as a degree of metal enrichment in the soil: CF = Cs/Cb. CF was classified into four groups [27,28] in CF < 1, no metal enrichment; 1 ≤ CF ≤ 3, moderate contamination; 3 ≤ CF ≤ 6, considerable contamination; CF > 6 very high contamination.

Ecological risk factor (Er) is quantitatively calculated to express the potential ecological risk with equitation suggested by Håkanson [27].

(1) $$E_{r}=T_{i}\cdot C_{f}$$

(2) $$C_{f}=C_{i}/B_{i}$$

where Ti is the toxic-response factor for a given substance, and Cf is the contamination factor. The Ti values of heavy metals by Håkanson [27] are given in Table 2. Ci is the metal concentration in an analyzed soil sample, and Bi is the reference value and could be used for some of the suggested values because it is not a uniform value, such as the background level, average crust level, national criteria, baseline level, *etc.* [26]. To describe the ecological risk factor the following terminology was used: Er < 40, low; 40 ≤ Er < 80, moderate; 80 ≤ Er < 160, considerable; 160 ≤ Er < 320, high; and Er ≥ 320, very high. The risk factor was used as a diagnostic tool for water pollution control, but it was also successfully used for assessing the contamination of soils in the environment by heavy metals.

The potential ecological risk index (RI) is defined as a sum of the risk factors (Equation (3)). Hakanson [27] and Yang [29] suggested RI represents heavy metals toxicity and environment response to all five risk factors (Pb, Cd, Cu, Zn, and Cr as total Cr) in playground soils. Many studies showed that the presence of toxic heavy metals can cause different type of health problems [30].

(3) $$\text{RI}=\sum E_{r}$$

To describe the RI, the following terminology was used: RI < 150, low risk; 150 ≤ RI < 300, moderate; 300 ≤ RI < 600, considerable; RI ≥ 600, very high.

Müller, in 1969, originally defined an index of geo-accumulation (Igeo) in order to define and determine metal contamination in soils [31] by comparing current concentrations with pre-industrial levels. The following equation can be used for calculation:

(4) $$I_{\text{geo}}=\log_{2}\left[\frac{C_{r}}{1.5\cdot C_{\text{ri}}}\right]$$

where Ci is the measured concentration of the examined metal in the sediment, and Cri is the geochemical background concentration, or reference value, of the metal i. Factor 1.5 is used because of possible variations in background values for a given metal in the environment, as well as very small anthropogenic influences. There are seven classes for the geo-accumulation index (Igeo), as determined by Müller [31]: Igeo ≤ 0, class 0, unpolluted; 0 <Igeo ≤ 1, class 1, from unpolluted to moderately polluted; 1 < Igeo ≤ 2, class 2, moderately polluted; 2 < Igeo ≤ 3, class 3, from moderately to strongly polluted; 3 < Igeo ≤ 4, class 4, strongly polluted; 4 < Igeo ≤ 5, class 5, from strongly to extremely polluted; and Igeo > 5, class 6, extremely polluted.

### 2.4. Statistical Analysis

Statistical data were obtained using SPSS statistical software 17.0 version. For the evaluation of correlation concentration coefficients for Pb, Cd, Cu, Zn, and Cr in soil samples Pearson’s correlation coefficient was used. Multivariate analysis using PCA was also performed.

## 3. Results and Discussion

### 3.1. Performance of the Analytical Procedure

Certified reference material IAEA 158 (sediment) and ERM-CC141 (loam soil) were used for checking the obtained data by determination of accuracy and precision. The recovery for all heavy metals Pb, Cd, Cu, Zn, and Cr ranged between 85%–110%. Precision as a relative standard deviation of triplicate measurement was less than 5% for all investigated elements. Standard reference materials were included in every batch of sample digestion and analysis as a part of the quality control protocol. Method validation showed lower detection limits and better sensitivity for Pb and Cd by GFAAS compared to ICP-OES. Also, the accuracy on certified reference materials was better for Pb and Cd on GFAAS.

The total contents and the descriptive statistics (minimum and maximum levels, as well as the means and standard deviations) for the five measured heavy metals that were investigated in the urban parks and playgrounds for this study are shown in Table 3, Table 4, Table 5, Table 6, Table 7, Table 8 and Table 9. The heavy metal distribution in the different soil samples showed spatial variations suggesting natural variability of concentrations and different origins of these elements. The order of the total element content was Zn > Cu > Pb > Cr > Cd. The mean content of all investigated elements did not exceed the maximum allowed concentration (MAC) values prescribed by National Regulation [32] (Table 3, Table 4, Table 5, Table 6, Table 7, Table 8 and Table 9), except for one location in Kotor (Table 9), which contributed to an increased mean Pb value. Content of Pb, Cd, Cu, and Zn at several locations was above MAC and background values suggested by Håkanson [27]. On several locations the concentrations of Pb were higher than MAC values (Table 6 and Table 9). Special attention should be paid to Pb concentrations because of its potential influence on human health [33]. The ingestion of contaminated soil or dust represents the main originating environmental source of Pb levels in the blood of children [34]. Monitoring of Pb content in soil is of great importance because of its negative effects on children’s central nervous systems and its contributions to developmental disorders, especially during long periods of exposure [35,36,37]. Harmful effects on blood, development, behavior, and intellectual functioning can be noticed as well as a result of ingestion of small quantities of Pb from dust or soil [37]. Increased risk of cancer development has also been associated to chronic Pb exposure [38].

### 3.2. Contamination Factor (CF)

The results of the CF for each measured element are presented in Table 10. The highest CF value, for Cu and Cd (considerable contamination), was found at site BR3. The CF values for Pb at KO7 location and CF for Zn at CT3 location showed moderate contamination. For Cr, there was no metal enrichment at any measured location. Mean CF values indicated no metal contamination for Pb, Cd, Zn, and Cr, and for Cu there was moderate contamination. Degree of metal enrichment could be set as follow Cu > Cd > Zn > Pb > Cr.

### 3.3. Ecological Risk Factor (Er)

The ecological risk factor results are shown in Table 10. For Zn, Cr, and Cu the potential Er indices were lower than 40. The Er value for Pb showed moderate ecological risk of pollution. Because of its higher toxicity coefficient, Cd presents a very high ecological risk in comparison to any of the other elements.

### 3.4. Ecological Risk Index (RI)

**Figure 2:**
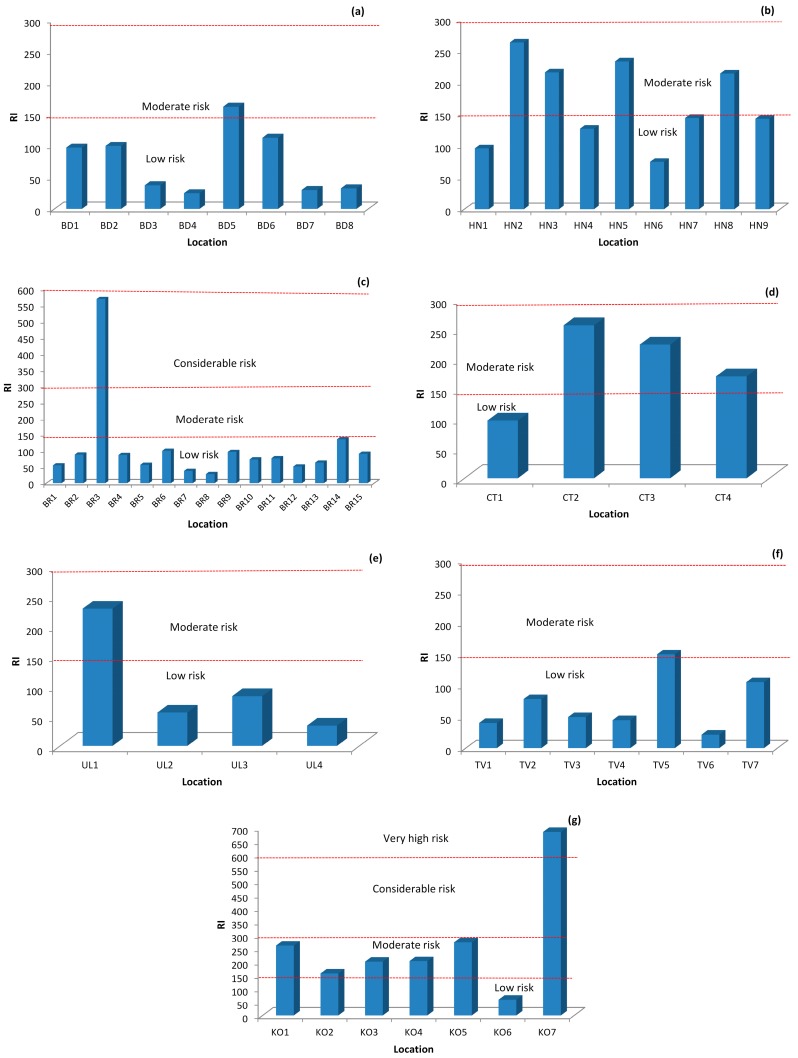
Ecological risk index (RI) values of heavy metals in urban parks soil samples in coastal municipalities of Montenegro, (**a**) Budva; (**b**) Herceg Novi; (**c**) Bar; (**d**) Cetinje; (**e**) Ulcinj; (**f**) Tivat; (**g**) Kotor.

Results for RI are presented in Figure 2. Generally, all RI values were lower than 300—even 150—what is in accordance to calculated Er (low to moderate risk). These results suggest soil samples had low and moderate ecological risks. Only one location, KO7, of the 54 soil samples showed an RI higher than 600, which points to a very high ecological risk of all elements. The main contributor to the RI is from the most toxic element, Cd, then Pb and Cu. Pollution from Cd has a long accumulation history and can represent a very strong ecological risk to both ecosystems and human health. In parks, playgrounds, and kindergartens, urban soils can influence children’s health due to their higher sensitivity; children are at a higher risk of exposure to the toxic metals than adults [39]. The body burden of Cd and Pb have been well documented as toxic to the central nervous [40,41] and renal systems [42]. Kidneys are the main target organ for the cumulative toxic metal exposure to Cd [43]. Copper (Cu) is a very important essential microelement, but can be unsafe when exposed to at higher doses. Chronic exposure to Cu dust or soil could cause health problems such as: nausea, head-aches, and diarrhea. In comparing data from this study with some previous studies from this region [4,5,7,8,9] it could be concluded that the potential influence of heavy metals to children’s health is at the minimum level for the investigated locations in Montenegro. Mean values of concentrations of all five metals are lower than MAC, and in some cases are much lower in comparison to cities in the region (*i.e.*, the Mediterranean region). 

### 3.5. Index of Geoaccumulation (Igeo)

**Figure 3:**
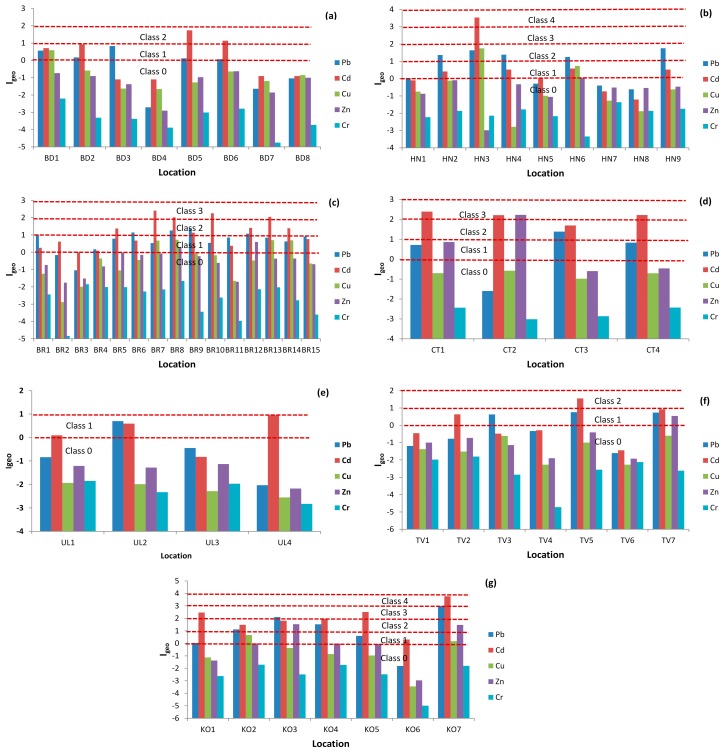
Geo-accumulation index (Igeo) values of heavy metals in urban parks soil samples in coastal municipalities of Montenegro, (**a**) Budva; (**b**) Herceg Novi; (**c**) Bar; (**d**) Cetinje; (**e**) Ulcinj; (**f**) Tivat; (**g**) Kotor.

According to the Igeo, most of the samples and elements belong to Class 0 and Class 2 (Table 10 and Figure 3) (*i.e.*, unpolluted and moderately polluted). Almost one third of locations belong to Class 1 (*i.e.,* unpolluted to moderately polluted soil).

The greatest contribution to Class 2 resulted from Pb (locations in Bar and Herceg Novi) and Cd concentrations (two locations in Budva and Kotor, one location in Tivat, and three locations in Herceg Novi). Class 3 (*i.e.,* from moderately to strongly polluted) contamination resulted from Cd concentrations (three locations in Cetinje, one location in Herceg Novi one, and two locations in Kotor). Class 4 (*i.e.,* strongly polluted), contamination resulted from Cd, Pb, and Cu (BR10 and KO7). All results indicate that the main contaminates are Cd and Pb and could be in following order Cd > Pb > Cu > Zn > Cr (Figure 3).

The lowest weight gives Cr = 0.474. PCA results are shown in Table 11 and Table 12. Five variables were subjected to an analysis of the main components. Prior to implementation of the PCA, the suitability of data for factor analysis was assessed. By examining the correlation matrix, it was discovered that a lot of coefficient values were 0.3 and higher.

The value of the Kaiser-Meyer-Oclyn indicator was 0.719, which exceeds the recommended value of 0.6 [44,45]. Bartlett’s test of sphericity [46] reached statistical significance; all of this indicates an adequate factorability correlation matrix. Principal component analysis revealed the presence of a component with a characteristic value of over 1, which explains 52.506% of the variance. By examining a past diagram, it established the existence of a clear point of fracture shown by the first component. Using Catell’s scree test, it wasdecided to retain displayed component for further investigation. This was further supported bz the results of Parallel Analysis, with eigenvalues exceedings the corresponding criterion values for a randomly generated data matrix of the same siye (5 variables × 54 respondents). This first principal component (PC1), explains 52.506% of the variance. The variables that give significant weight selected components are Pb = 0.874; Cd = 0.873; Zn = 0.686; Cu = 0.636). This may indicate that the urban soils are under influence of anthropogenic inputs of these elements. In the present study, in addition to traffic emissions, this metal contamination may be linked to touristic pollution.

To analyze the relationships among metal concentrations, a Pearson’s correlation analysis was applied, and the results are presented in Table 13. Based on data shown in Table 13, Pb, Cd, Cu, and Zn were significantly positively correlated with each other; Cd and Pb (*r* = 0.731), Cu and Pb (*r* = 0.388), Cu and Cd (*r* = 0.521), Zn and Pb (*r* = 0.555), Zn and Cd = (0.494 ) at a significance level of 0.01. Generally Pb, Cd, Cu, and Zn were highly correlated with each other, indicating that primarily anthropogenic sources such as traffic and industrial activities contribute to contamination [4,47,48,49]. A significant weak positive correlation is found between Cr and Pb (*r* = 0.327, *p* < 0.05), indicating that Cr had some different origin, probably natural.

## 4. Conclusions

Based on the obtained data, mean CF values indicated no metal contamination for Pb, Cd, Zn, and Cr, and for Cu there was moderate contamination. For Zn, Cr, and Cu the potential Er indices were low, for Pb moderate, but for Cd very high on account of two locations that contributed to the total Er being very high. The rest of the data for all the locations showed low ecological risk for pollution. Based on the result for RI, it can be concluded that almost all investigated soil samples showed low to moderate pollution. The main contributor to RI is from the most toxic element, Cd (what is in accordance with Er values), then Pb and Cu. Based on RI values for each municipality it could be concluded that some locations in Bar and Kotor are under heavy metal contamination pressure. These locations represent potential risks to children’s health because RI values for heavy metals are from a considerable to very high risk. Pollution from Cd has a long accumulation history and can represent very strong ecological risks to both ecosystems and human health. According to the Igeo, most of samples and elements belong to unpolluted to moderately polluted soils. All results indicate that the main contaminates are Cd and Pb. The number of people in this area increases dramatically during tourist seasons, and this is what generally contributes to the pollution in city parks. Increases of Pb and Cd concentrations were noticed, and this is what indicates that the urban soils in the parks and playgrounds in the cities have been significantly impacted by heavy metals derived from anthropogenic activities. PCA analysis showed that the main contributor to soil pollution is Pb. Pearson’s correlation coefficient showed that Cd and Pb originated from anthropogenic pollution, Cu and Zn from natural sources, and Cr has a lithogenic origin. This data provides enough evidence about soil pollution at some locations in coastal municipalities of Montenegro, and it is well documented that the presence of highly toxic heavy metals in the environment can cause various types of health problems. Based on this new data of soil contamination in Montenegro, it is recommended that continuous research be performed and that this data is used for the calculation of the carcinogen and non-carcinogen Hazard Index for children as well as for adults. This kind of studies provides some early warning signals about heavy metal pollution in soils. Special attention should be paid to this problem, further research should be continued, and possible ways of site remediation should be considered where contamination has been observed. For improving ecological risk assessment and management of trace elements in soil samples, the calculation of pollution indices and the application of statistical methods is recognized as a useful tool to reduce pollutant emission and minimize the hazard risks to human health.

## Figures and Tables

**Table 1:** Coordinates, area and soil types of sampling sites.

Location Site	Coordinates	Area (km^2^)	Soil Type National/World Reference Base
Budva	42°17’17’’N	122	Eutric cambisol/Eutric cambisol
	18°50’33’’E		Fluvisol/Fluvisols
Herceg Novi	42°27’10.62’’N	235	Eutric cambisol/Eutric cambisol
	18°31’52.33’’E		Fluvisol/Fluvisols
Tivat	42.43°N	46	Cambisol/Cambic Umbrisols
	18.70’E		
Bar	42.10°N	598	Eutric cambisol/Eutric cambisol
	19.10°E		Fluvisol/Fluvisols
Cetinje	42.38°N	910	Rendzina/Rendzic Leptosols
	18.92°E		
Ulcinj	41.92°N	255	Eutric cambisol/Eutric cambisol
	19.20’E		
Kotor	42°25’48’’N	335	Cambisol/Cambic Umbrisols
	18°46’12’’E		Fluvisol/Fluvisols

**Table 2:** Toxic-response factor by Håkanson [27].

Elements	Cd	Cu	Pb	Cr	Zn
**Toxic-response factor**	30	5	5	2	1

**Table 3:** Total contents and descriptive statistics of elements in Budva (BD) urban parks soil samples, this study (mg⋅kg^−1^).

Sampling Site	Element				
	Pb	Cd	Cu	Zn	Cr
BD1	27.68	0.49	124.06	62.87	32.51
BD2	21.14	0.57	54.91	56.00	15.04
BD3	33.30	0.14	26.72	40.39	14.40
BD4	2.86	0.14	26.11	14.02	10.12
BD5	20.35	1.00	34.17	53.40	18.56
BD6	19.71	0.66	52.93	67.88	21.69
BD7	6.02	0.16	36.01	28.95	5.55
BD8	9.12	0.16	45.78	52.31	11.31
MAC	50	2	100	300	50
Min	2.86	0.14	26.11	14.02	5.55
Max	33.30	1.00	124.06	67.88	32.51
Mean	17.48	0.42	50.09	46.98	16.77
Mediana	20.06	0.33	40.90	52.86	15.08
SD *	10.61	0.32	31.84	18.108	7.95

* Standard deviation.

**Table 4:** Total contents and descriptive statistics of elements in Herceg Novi (HN) urban parks soil samples for this study (mg⋅kg^−1^).

Sampling Site	Element				
	Pb	Cd	Cu	Zn	Cr
HN1	41.59	0.48	60.67	94.21	31.08
HN2	27.10	1.59	131.94	100.06	33.85
HN3	44.88	1.22	136.51	129.4	47.38
HN4	49.07	0.66	78.44	89.64	13.86
HN5	27.39	1.43	73.33	68.76	24.42
HN6	33.78	0.39	26.33	32.29	9.64
HN7	39.49	0.80	59.30	159.17	34.10
HN8	33.70	1.25	134.15	81.64	36.88
HN9	28.98	0.78	132.76	81.75	21.91
MAC	50	2	100	300	50
Min	27.10	0.39	26.33	32.29	9.64
Max	49.07	1.59	136.51	159.17	47.38
Mean	35.55	1.12	96.59	92.84	28.59
Mediana	33.74	1.01	105.19	85.69	33.85
SD *	8.22	0.42	42.69	38.35	13.37

* Standard deviation.

**Table 5:** Total contents and descriptive statistics of elements in Tivat (TV) urban parks soil samples for this study (mg⋅kg^−1^).

Sampling Site	Element				
	Pb	Cd	Cu	Zn	Cr
TV1	8.17	0.22	31.66	52.38	37.85
TV2	10.94	0.47	28.76	63.14	42.82
TV3	28.91	0.21	53.84	47.45	20.85
TV4	14.89	0.25	17.15	28.02	5.67
TV5	31.66	0.88	41.09	78.81	25.46
TV6	6.15	0.11	17.13	27.54	34.35
TV7	31.19	0.57	54.16	152.82	24.41
MAC	50	2	100	300	50
Min	6.15	0.11	17.13	27.54	5.67
Max	31.66	0.88	54.16	152.82	42.82
Mean	18.84	0.39	34.83	64.31	27.34
Mediana	14.89	0.25	31.66	52.38	25.46
SD *	11.34	0.27	15.53	43.09	12.40

* Standard deviation.

**Table 6:** Total contents and descriptive statistics of elements in Bar (BR) urban parks soil samples for this study (mg⋅kg^−1^).

Sampling Site	Element				
	Pb	Cd	Cu	Zn	Cr
BR1	18.84	0.28	49.03	57.20	31.88
BR2	48.34	0.40	76.08	98.41	40.99
BR3	58.23	3.47	277.4	13.26	34.04
BR4	48.84	0.43	11.97	83.63	43.71
BR5	15.35	0.31	41.45	50.54	33.29
BR6	47.00	0.45	137.20	108.27	14.72
BR7	14.23	0.18	34.17	73.42	58.34
BR8	12.23	0.13	22.38	72.21	41.20
BR9	63.33	0.43	53.56	75.92	44.77
BR10	37.77	0.36	34.92	63.28	27.64
BR11	16.80	0.46	11.22	31.02	5.24
BR12	9.12	0.30	20.84	36.64	41.64
BR13	21.15	0.32	64.34	59.70	37.26
BR14	32.34	0.78	40.03	103.79	37.05
BR15	4.60	0.58	14.10	23.28	21.16
MAC	50	2	100	300	50
Min	4.6	0.18	11.22	13.26	5.24
Max	63.33	3.47	277.40	108.27	58.34
Mean	29.88	0.63	59.23	63.37	34.19
Mediana	21.15	0.42	40.03	63.28	37.05
SD *	19.26	0.83	68.37	28.93	13.07

* Standard deviation.

**Table 7:** Total contents and descriptive statistics of elements in Cetinje (CT) urban parks soil samples for this study (mg⋅kg^−1^).

Sampling Site	Element				
	Pb	Cd	Cu	Zn	Cr
CT1	35.26	0.51	52.73	65.09	12.34
CT2	30.82	1.57	50.70	190.47	27.66
CT3	6.18	1.39	55.30	491.55	18.53
CT4	48.90	0.97	41.74	69.25	20.57
MAC	50	2	100	300	50
Min	6.18	0.51	41.74	65.09	12.34
Max	48.90	1.57	55.30	491.55	27.66
Mean	30.29	1.11	50.12	204.09	19.78
Mediana	33.04	1.18	51.72	129.86	19.55
SD *	17.82	0.47	5.89	200.27	6.31

* Standard deviation.

**Table 8:** Total contents and descriptive statistics of elements in Ulcinj (UL) urban parks soil samples for this study (mg⋅kg^−1^).

Sampling Site	Element				
	Pb	Cd	Cu	Zn	Cr
UL1	33.28	1.39	50.43	76.24	27.82
UL2	10.47	0.32	21.60	45.53	41.73
UL3	30.42	0.45	20.84	43.38	29.86
UL4	13.73	0.17	16.97	48.09	38.33
MAC	50	2	100	300	50
Min	10.47	0.17	16.97	43.38	27.82
Max	33.28	1.39	50.43	76.24	41.73
Mean	21.98	0.58	27.46	53.31	34.44
Mediana	22.08	0.39	21.22	46.81	34.10
SD *	11.54	0.55	15.45	15.41	6.66

* Standard deviation.

**Table 9:** Total contents and descriptive statistics of elements in Kotor (KO) urban parks soil samples for this study (mg⋅kg^−1^).

Sampling Site	Element				
	Pb	Cd	Cu	Zn	Cr
KO1	19.18	1.66	37.70	40.31	24.38
KO2	40.61	0.84	130.91	103.97	45.82
KO3	80.31	1.05	63.90	303.90	26.65
KO4	54.07	1.16	45.37	103.89	45.21
KO5	28.37	1.70	42.05	100.96	26.89
KO6	5.32	0.37	7.57	13.44	4.72
KO7	148.40	4.08	92.84	291.42	43.01
MAC	50	2	100	300	50
Min	5.32	0.37	7.57	13.44	4.72
Max	148.40	4.08	130.91	303.90	45.82
Mean	53.75	1.55	60.05	136.84	30.95
Mediana	40.61	1.16	45.37	103.89	26.89
SD *	48.34	1.21	40.63	115.33	14.92

* Standard deviation.

**Table 10:** Contamination factor (CF), ecological risk factor (Er), geo-accumulation index (Igeo) and ecological risk index (RI) values of heavy metals in soil.

Descriptive Statistics	Element				
	Pb	Cd	Cu	Zn	Cr
CF					
Min	0.04	0.11	0.15	0.08	0.01
Max	2.13	3.47	5.55	2.81	0.65
Mean	0.43	0.76	1.16	0.49	0.63
Er					
Min	1.15	16.5	0.682	0.19	0.10
Max	59.52	520.5	25.2	7.02	1.17
Mean	12.08	114.3	5.25	1.22	0.56
Igeo					
Min	<0	<0	<0	<0	<0
Max	2.98	3.76	1.75	2.23	<0
Mean	0.29	0.83	<0	<0	<0

**Table 11:** Total variance explained.

Component	Initial Eigen Values			Extraction Sums of Squared Loadings		
	Total	% of Variance	Cumulative %	Total	% of Variance	Cumulative %
**Pb**	2.625	52.506	52.506	2.625	52.506	52.506
**Cd**	0.896	17.919	70.424			
**Cu**	0.805	16.103	86.528			
**Zn**	0.434	8.680	95.208			
Cr	0.240	4.792	100.000			

**Table 12:** Principal component analysis (PCA).

Component Matrix	
	PC1
Pb	0.874
Cd	0.873
Zn	0.686
Cu	0.636
Cr	0.474

**Table 13:** Correlation coefficients between different metals (*n* = 54).

Component	Pb	Cd	Cu	Zn	Cr
Pb	1				
Cd	0.731 **				
Cu	0.388 **	0.521 **	1		
Zn	0.555 **	0.494 **	0.178	1	
Cr	0.327 *	0.231	0.246	0.184	1

** Correlation is significant at the 0.01 level (2-tailed). * Correlation is significant at the 0.05 level (2-tailed).
